# Increasing prevalence of infectious diseases in asylum seekers at a tertiary care hospital in Switzerland

**DOI:** 10.1371/journal.pone.0179537

**Published:** 2017-06-15

**Authors:** Constantine Bloch-Infanger, Veronika Bättig, Jürg Kremo, Andreas F. Widmer, Adrian Egli, Roland Bingisser, Manuel Battegay, Stefan Erb

**Affiliations:** 1 Division of Infectious Diseases & Hospital Epidemiology, University Hospital Basel, University Basel, Basel, Switzerland; 2 Departement of Internal Medicine, Kantonsspital Uri, Altdorf, Switzerland; 3 Private Medical Office, Basel, Switzerland; 4 Division of Clinical Microbiology, University Hospital Basel, University Basel, Basel, Switzerland; 5 Departement of Emergency, University Hospital Basel, University Basel, Basel, Switzerland; National Institute for Health and Welfare, FINLAND

## Abstract

**Objective:**

The increasing number of refugees seeking asylum in Europe in recent years poses new challenges for the healthcare systems in the destination countries. The goal of the study was to describe the evolution of medical problems of asylum seekers at a tertiary care centre in Switzerland.

**Methods:**

At the University Hospital Basel, we compared all asylum seekers during two 1-year time periods in 2004/05 and 2014/15 concerning demographic characteristics and reasons for referrals and hospitalizations.

**Results:**

Hundred ninety five of 2’544 and 516 of 6’243 asylum seekers registered at the national asylum reception and procedure centre Basel were referred to the University Hospital Basel in 2004/05 and 2014/15, and originated mainly from Europe (62.3%, mainly Turkey) and Africa (49.1%, mainly Eritrea), respectively. Median age was similar in both study periods (26.9 and 26.2 years). Infectious diseases in asylum seekers increased from 22.6% to 36.6% (p<0.001) and were the main reasons for hospitalizations (33.3% of 45 and 55.6% of 81 hospitalized patients, p = 0.017) in 2004/05 compared to 2014/15. The leading infectious diseases in hospitalized patients were tuberculosis (n = 4) and bacterial skin infections (n = 2) in 2004/05; Malaria (n = 9), pneumonia (n = 6), Chickenpox (n = 5), other viral infections (n = 5) and bacterial skin infections (n = 5) in 2014/15. Infectious diseases like malaria, cutaneous diphtheria, louseborne-relapsing fever or scabies were only found in the second study period. Almost one third of the admitted asylum seekers required isolation precautions with median duration of 6–9.5 days in both study periods.

**Conclusions:**

The changing demography of asylum seekers arriving in Switzerland in the current refugee crisis has led to a shift in disease patterns with an increase of infectious diseases and the re-emergence of migration-associated neglected infections. Physicians should be aware of these new challenges.

## Introduction

By the end of 2014, almost 60 million people were displaced due to conflicts, persecution or repressions worldwide [1]. This is the highest number since the end of World War II, and the highest annual increase observed in a one year period. In 2015, approximately 1.6 million refugees sought for asylum in Europe, which was an increase of approximately 80% compared to 2014. In Switzerland asylum applications peaked to 39’523 in 2015 [2]. The most important countries of origin of asylum seekers in Switzerland in 2015 were Eritrea, Syria and Afghanistan, representing more than 65% of all applications [3,4]. The increase in refugees arriving to Europe poses a challenge not only with regard to accommodation, social services, politics and logistical problems like timely processing of asylum applications, but also to the health care systems of the destination countries.

Knowledge about health needs and the frequency of communicable diseases in asylum seekers is evolving but still limited [5]. According to studies conducted in the last 10 years, main health problems of asylum seekers were chronic diseases, psychiatric conditions such as post-traumatic stress disorder or depression, and infectious diseases [6] [7] [8] [9]. In recent studies from Switzerland the prevalence of infectious diseases seemed to be much lower than expected depending mainly on the region of origin of the refugees [8] [10] [11].

The current increase in refugees and the shift in countries of origin of asylum seekers compared to former years are likely to influence the type and frequency of medical problems including infectious diseases. The goal of our study was to describe the major changes in demographic characteristics, reasons for hospital referrals and hospitalizations inclusive infectious diseases, length of hospital stay, and the requirement of isolation precautions in asylum seekers presenting at the University Hospital Basel, Switzerland in two one-year time periods ten years apart.

## Methods

### Study setting and design

Refugees arriving to Switzerland can apply for asylum at one of the five federal reception and procedure centres, one of which is located in the Canton of Basel-Stadt. All asylum seekers receive a healthcare insurance by the Federal Office of Migration, which covers the costs of all basic medical services. Informations about the Swiss healthcare system, the possibility of vaccination and HIV/AIDS are provided. Within five days from arrival at the reception and procedure centre, all asylum seekers are screened for tuberculosis by a nurse with a standardized audiovisual questionnaire in 28 languages. No other systematic screening examinations for other diseases like e.g. HIV or hepatitis B are performed. Asylum seekers with conspicuous results in the questionnaire and/or with an acute health problem are assessed by a dedicated primary care physician acting as a general practitioner. If the health problem cannot be solved on-site, the asylum seeker is usually referred to the multidisciplinary Emergency Department of the University Hospital of Basel, a 900-bed tertiary care centre. Asylum seekers with psychiatric problems are usually referred to the Psychiatric University Hospital of Basel and children < 16 years to the University Children’s Hospital of Basel.

Of note, prior to 2006 the tuberculosis screening was done by a chest-X-ray for all asylum seekers arriving at the Swiss border [12].

For this study, one-year data from two different time periods 10 years apart, from 1^st^ September 2004 until 31^st^ August 2005 (= 2004/05) and 1^st^ September 2014 until 31^st^ August 2015 (= 2014/15), were collected for all adult asylum seekers with an age ≥16 presenting to the University Hospital of Basel.

### Data collection and data analysis

Data for this study were collected retrospectively from the hospital’s electronic health records, and detailed chart review was performed. Electronic health records contain demographic data such as sex, age and country of origin, reason for presentation, diagnosis, therapies, isolation precautions, and discharge reports. The countries of origin were grouped according to the six WHO regions: African Region (AFR), Region of the Americas (AMR), South-East Asia Region (SEAR), European Region (EUR), Eastern Mediterranean Region (EMR) and Western Pacific Region (WPR) (http://www.who.int/about/regions).

The patients diagnoses were grouped according to the written final diagnoses. An infectious disease was defined as any disorder which was 1) caused by microbiologically confirmed pathogenic microorganisms, such as bacteria, viruses, parasites or fungi, 2) clinically suggestive for an infectious disease or 3) treated with antimicrobial agents. The remaining patients were grouped into internal medicine, gynaecology and obstetrics, neurology, surgery, psychiatrics and oncology.

Data between the two time periods 2004/05 and 2014/15 were compared. Univariable analysis was performed using the χ2 test or Fisher’s exact test, where applicable for categorical variables. Continuous variables were compared using 2-tailed Student’s t test or Mann-Whitney U test in cases of nonparametric data. Two-tailed p-values of <0.05 were considered statistically significant. Statistical analysis was done with IBM SPSS Statistics for Windows, Version 22.0 Armonk, NY, USA.

### Ethical considerations

The study (study ethic number 2015–00166) was approved by the local Ethics Committee, the Ethikkommission Nordwest- und Zentralschweiz, EKNZ, Switzerland. In accordance with the requirements of the Ethics Committee, patient information was anonymized and de-identified prior to analysis.

## Results

### Patient characteristics

**Fig 1:**
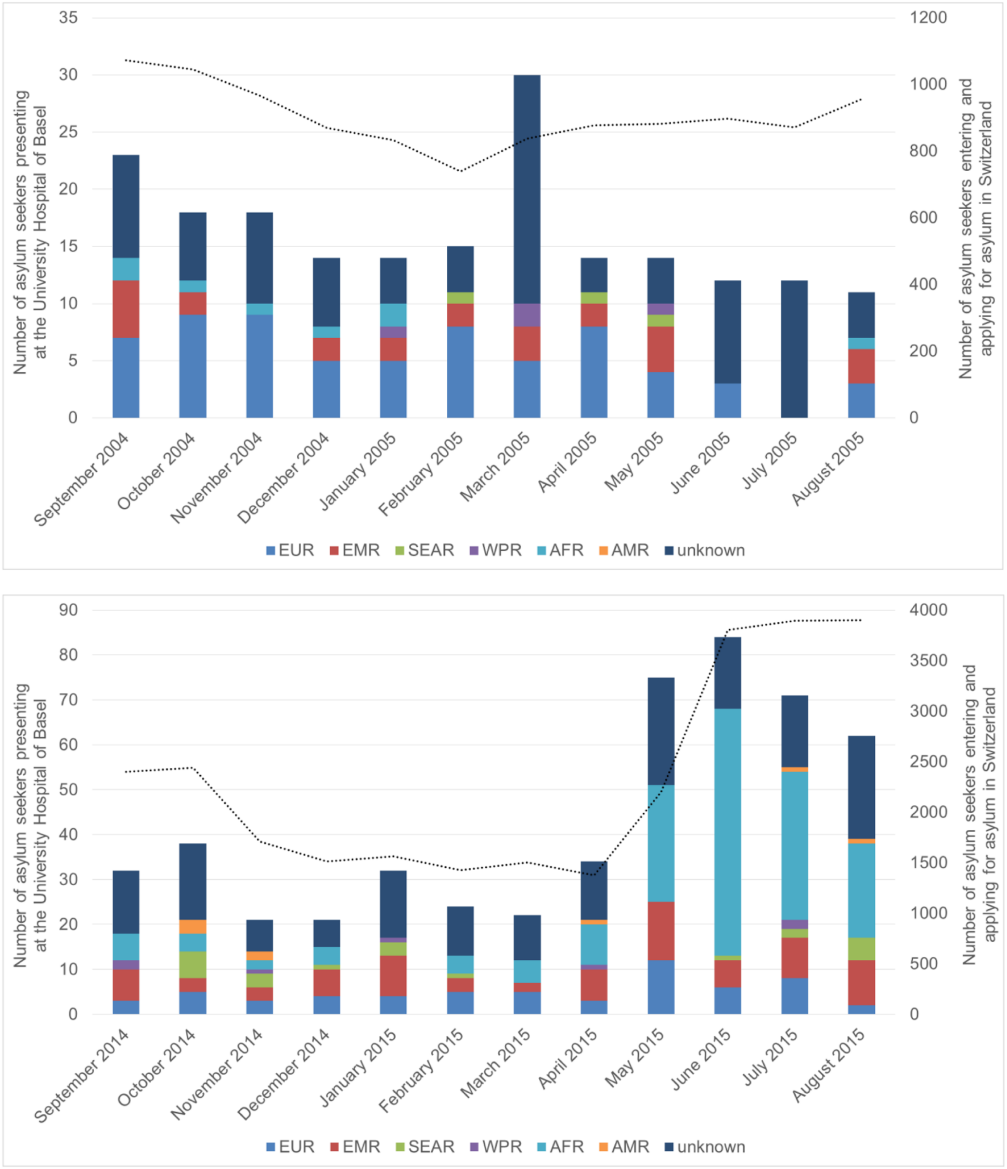
Monthly number of asylum seekers referred to the University Hospital Basel stratified for region of origin. (A) time period 2004/05 (B) time period 2014/15. AFR: African Region; AMR: Region of the Americas; EMR: Eastern Mediterranean Region; EUR: European Region; SEAR: South-East Asia Region; WPR: Western Pacific Region; unknown: no data about the region of origin available. The bars represent the number of asylum seekers presenting at the University Hospital Basel per month and stratified into region of origin. Corresponding y-axis on the left of the diagram (n = 0–35 and 0–90, respectively). Black dotted line represents the monthly number of all asylum seekers entering Switzerland and applying asylum at any of the 5 national reception and procedure centres. Corresponding y-axis on the right of the diagram (n = 0–1200 and 0–4000, respectively).

A total of 8’697 asylum seekers were registered at the national asylum reception and procedure centre Basel during both study periods: 2’454 asylum seekers in 2004/05 and 6’243 in 2014/15. Average stay at the local asylum reception and procedure centre was 20.1 and 28.4 days in 2004/05 and 2014/15, respectively (S1 Table). 195 (7.9%) were referred to the University Hospital Basel in 2004/05. Amongst them 45 (23.1%) asylum seekers had to be hospitalized. In 2014/15 516 asylum seekers (8.3%) were referred to the University Hospital Basel and 81 (15.7%) were hospitalized. The evolution of monthly referrals to the University Hospital Basel during the two study periods is shown in Fig 1.

Patient characteristics of all referred patients and those hospitalized at the University Hospital Basel are described in Table 1. Patients in both study periods were mainly male, however male predominance was less pronounced in the second study period (70.3% in 2004/05 versus 57.8% in 2014/15). Median age was similar in both study periods (26.9 years (IQR 22.9–36.9) and 26.2 years (IQR 20.7–35.5), respectively). In 2004/05 most asylum seekers originate from the European region (62.3% of patients with known origin) whereas in 2014/15 it was the African Region (49.1% of patients with known origin). The 5 most frequent countries of origin in 2004/05 were Turkey (n = 27, 25.5% of patients with known origin), Bulgaria (n = 15, 14.2%), Iraq (n = 12, 6.2%), Georgia (n = 6, 5.7%) and Nigeria (n = 5, 4.7%); in 2014/15 Eritrea (n = 154, 44.8% of patients with known origin), Syria (n = 22, 6.4%), Sri Lanka (n = 21, 6.1%), Kosovo (n = 15, 4.4%) and Somalia (n = 15, 4.4%) (S1 and S2 Tables). In the subgroup of the hospitalized patients, a similar distribution in countries of origin was observed.

**Table 1:** Patient characteristics of all referred and hospitalized patients at the University Hospital Basel during the two study periods 2004/05 and 2014/15.

	2004/05				2014/15				p-value
	All patients n = 195		Hospitalized patients n = 45 (23.1%)		All patients n = 516		Hospitalized patients n = 81 (15.7%)		
**Demographic characteristics (n, % or median, IQR)**									
Male	137	70.3%	31	68.9%	298	57.8%	53	65.4%	0.003*
Female	58	29.7%	14	31.1%	218	42.2%	28	34.6%	
Age in years	26.9	22.9–36.9	26.8	20.6–35.5	26.2	20.7–35.5	26.5	20.9–35.8	0.34*
**Region of origin (n, %)**									
EUR	66	33.8%	25	55.6%	60	11.6%	13	16.0%	<0.0001*
EMR	25	12.8%	7	15.6%	78	15.1%	12	14.8%	0.439*
SEAR	3	1.5%	0	0.0%	22	4.3%	4	4.9%	0.078*
WPR	4	2.1%	2	4.4%	7	1.4%	3	3.7%	0.736*
AFR	8	4.1%	2	4.4%	169	32.8%	44	54.3%	<0.0001*
AMR	0	0.0%	0	0.0%	8	1.6%	3	3.7%	
Unknown	89	45.6%	9	20.0%	172	33.3%	2	2.5%	0.003*
**Reasons for referral (n, %)**									
Infectious disease	44	22.6%	15	33.3%	189	36.6%	45	55.6%	<0.001*
Non-infectious disease	151	77.4%	30	66.7%	327	63.4%	36	44.4%	
**Reasons for hospital admissions (n, %)**									
Infectious diseases			15	33.3%			45	55.6%	0.02
Internal medicine			5	11.1%			15	18.5%	0.23
Gynaecology and obstetrics			11	24.4%			7	8.6%	0.02
Neurology			2	4.4%			7	8.6%	0.49
Surgery			7	15.6%			3	3.7%	0.03
Psychiatrics			4	8.9%			2	2.5%	0.19
Oncology			1	2.2%			2	2.5%	1.00
**Duration of hospitalization in days (median, IQR)**									
All hospitalized patients			6	5–11			5	4–10	0.28
Patients with infectious diseases			7	5–20			7	4–10	0.41
Patients with non- infectious diseases			6	4–9			4.5	2–6	0.14
**Isolation (n, % or median, IQR)**									
Patients isolated			13	28.9%			24	29.6%	0.920
Duration of isolation in days			9.5	5–21			6	3–10	0.21

AFR: African Region; AMR: Region of the Americas; EMR: Eastern Mediterranean Region; EUR: European Region; SEAR: South-East Asia Region; WPR: Western Pacific Region; IQR: interquartile range

* p-values refer to comparisons of variables from all patients between the two study periods

### Reasons for hospital referrals and admissions

During the first study period 2004/05 22.6% of all referred and 33.3% of the hospitalized patients had an infectious disease. Ten years later in 2014/15, this number increased to 36.6% for all patients (p<0.001) and 55.6% for the hospitalized patients (p = 0.017).

**Fig 2:**
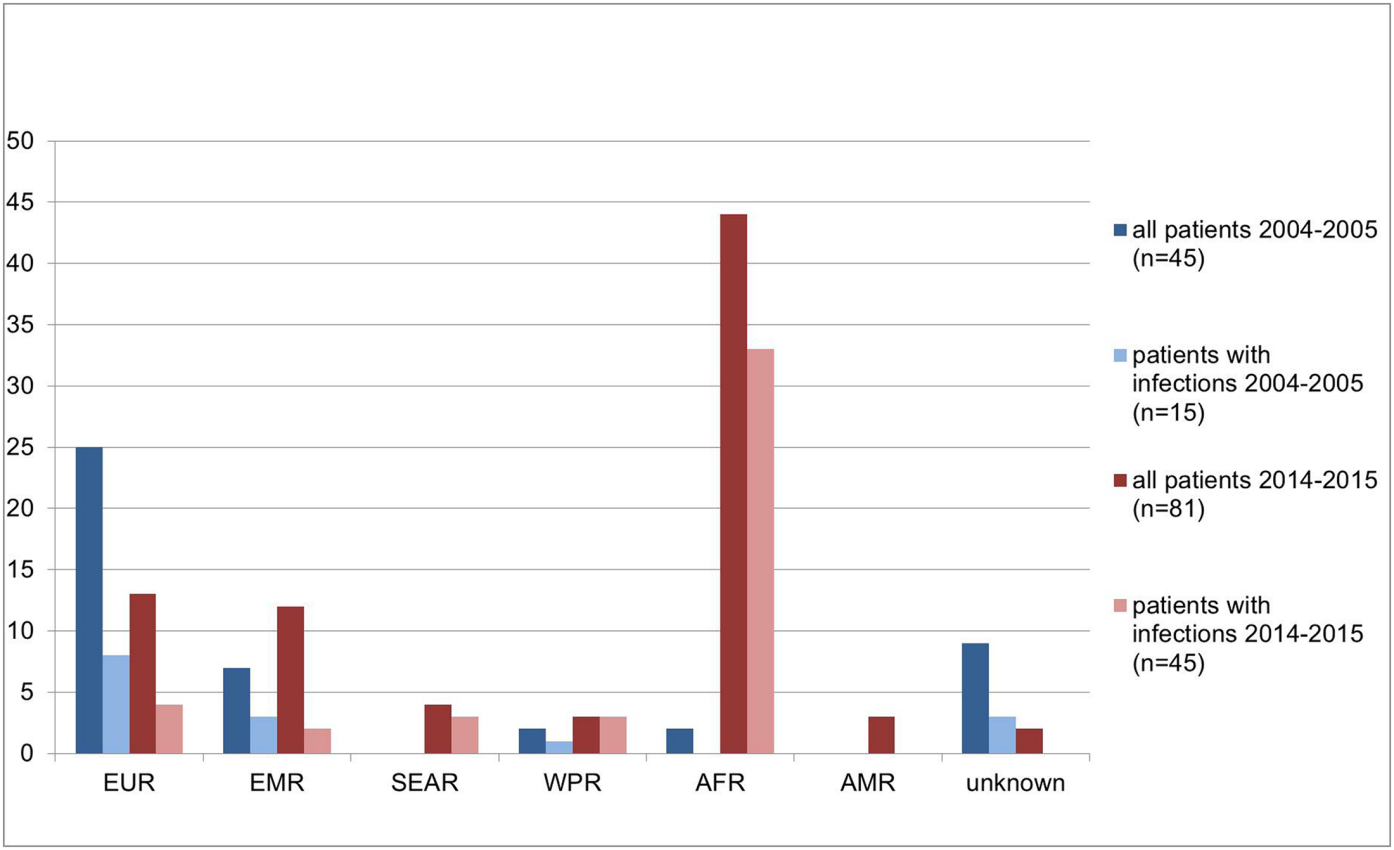
Comparison of all hospitalized patients (n = 126) and hospitalized patients with infections (n = 60) between both study periods stratified by region of origin. AFR: African Region; AMR: Region of the Americas; EMR: Eastern Mediterranean Region; EUR: European Region; SEAR: South-East Asia Region; WPR: Western Pacific Region.

Infectious diseases were in both study periods the main reasons for hospitalizations (Table 1) and accounted for more than 70% of hospital admissions in asylum seekers from the African, West Pacific and the South-East Asia Region in 2014/15 (Fig 2).

The leading infectious diseases in hospitalized asylum seekers were tuberculosis (n = 4) and bacterial skin infections (n = 2) in 2004/05; Malaria (n = 9), pneumonia (n = 6), Chickenpox (n = 5), other viral infections (n = 5), bacterial skin infections (n = 5), ear-nose-throat infections (n = 2), influenza (n = 3) and tuberculosie (n = 2) in 2014/15 (Table 2). Notably, in the second study period 2014/15 a significant proportion of patients presented with tropical infectious diseases such as malaria, furthermore re-emerging infectious diseases such as louseborne relapsing fever (n = 2) and cutaneous diphtheria (n = 3) were diagnosed.

**Table 2:** Diagnosis of all hospitalized patients with infectious diseases and their region of origin during the two study periods.

Diagnoses	n*	% of all hospitalized patients^$^	% of hospita-lized patients with infection	Regions of origin
**2004/05**	n = 15			
Tuberculosis	4	8.9%	50.0%	EMR (n = 1), EUR (n = 2), unknown (n = 1)
Bacterial skin infection	2	4.4%	25.0%	EMR (n = 1), unknown (n = 1)
Atypical pulmonary mycobacteriosis	1	2.2%	12.5%	EUR (n = 1)
Viral pulmonary infection	1	2.2%	12.5%	unknown (n = 1)
Exclusion of tuberculosis	7	15.6%	-	EMR (n = 1), EUR (n = 5), WPR (n = 1)
**2014/15**	n = 45			
Malaria (8 M. vivax, 1 M. falciparum)	9	11.1%	20.4%	AFR (n = 9)
Bacterial pulmonary infection	6	7.4%	13.6%	AFR (n = 4), EMR (n = 1), EUR (n = 1)
Chickenpox	5	6.2%	11.4%	AFR (n = 4), EMR (n = 1)
Viral infection not otherwise specified	5	6.2%	11.4%	AFR (n = 5)
Bacterial skin- and soft tissue infection	5	6.2%	11.4%	AFR (n = 5)
ENT infection	4	4.9%	9.1%	AFR (n = 2), EUR (n = 1), SEAR (n = 1)
Influenza	3	3.7%	6.9%	AFR (n = 1), EUR (n = 1), SEAR (n = 1)
Active tuberculosis	2	2.5%	4.5%	WPR (n = 2)
Louseborne relapsing fever	2	2.5%	4.5%	AFR (n = 2)
Septic arthritis	1	1.3%	2.3%	EUR (n = 1)
Meningococcal meningitis	1	1.3%	2.3%	SEAR (n = 1)
Fungal pulmonary infection	1	1.3%	2.3%	AFR (n = 1)
Exclusion of tuberculosis	1	1.3%	-	WPR (n = 1)

AFR: African Region; AMR: Region of the Americas; EMR: Eastern Mediterranean Region; EUR: European Region; SEAR: South-East Asia Region; WPR: Western Pacific Region; ENT: ear nose throat infection

* number of hospitalized patients with infection;

^$^ 45 patients hospitalized in 2004/05 and 81 patients hospitalized in 2014/15

During the second study period 12 patients originating mainly from the African Region presented with two or more infectious problems, mainly co-infections with scabies (n = 8), colonisation with MRSA (n = 3) and cutaneous diphtheria (n = 2) (S3 Table) Screening tests for HIV as well as hepatitis B and C were ordered frequently but not systematically, however only two new HIV diagnoses were noted both during the second study period.

### Isolation precautions

Thirteen of 45 (28.9%) and 24 of 81 (29.6%) hospitalized asylum seekers required isolation precautions in 2004/05 and 2014/15, respectively (Table 3).

**Table 3:** Reasons for isolation precautions of hospitalized asylum seekers during the two study periods.

	n*	% of hospitalized patients^$^	% of isolated patients
**2004/05**	n = 13	28.9%	100.0%
Suspected active tuberculosis (until exclusion of tuberculosis)	8	17.8%	61.5%
Confirmed tuberculosis	4	8.9%	30.8%
MRSA colonisation	1	2.2%	7.7%
**2014/15**	n = 24	29.6%	100.0%
Suspected active tuberculosis (until exclusion of tuberculosis)	9	11.1%	37.5%
Scabies	7	8.6%	29.2%
Chickenpox (primary varicella virus infection)	5	6.2%	20.8%
MRSA colonisation	4	4.9%	16.7%
Influenza	3	3.7%	12.5%
Cutaneous diphtheria	3	3.7%	12.5%
Confirmed tuberculosis	2	2.5%	8.3%
Meningococcal meningitis	1	1.2%	4.2%

MRSA: Methicillin-resistant *Staphylococcus aureus*

* number of hospitalized patients with infection;

^$^ 45 patients hospitalized in 2004/05 and 81 patients hospitalized in 2014/15

In 2004/05 most isolated asylum seekers came from the European Region (n = 7), followed by 4 from Eastern Mediterranean Region and one from Western Pacific Region and one was unknown. Twelve of 13 patients were isolated because of suspected active tuberculosis. Median duration of isolation was 9.5 days (range 1–30 days). Longest duration of isolation was found in two patients with active pulmonary tuberculosis (30 and 22 days).

In the second study period, the majority of the isolated patients came from the African Region (n = 16, 66.7%), followed by patients from the Western Pacific Region (n = 3, 12.5%), the Eastern Mediterranean Region (n = 2, 8.3%), the South-East Asia Region (n = 2, 8.3%), and the European Region (n = 1, 4.2%). In this study period, 8 patients had more than one reason to be isolated. 11 patients were isolated for suspected active tuberculosis, whereas tuberculosis could be confirmed in two patients and alternative diagnoses such as pneumonia and viral or fungal pulmonary infections were detected in 8 patients. Median duration of isolation was 6 days (range 1–61 days). Similar to the first study period, longer duration of isolation was found in the two patients with confirmed active pulmonary tuberculosis (16 and 61 days).

## Discussion

In this retrospective study investigating the reasons for referrals of asylum seekers to an urban tertiary hospital in Switzerland in 2004/05 and 2014/15, we found a significant shift in regions of origin and disease patterns over time. Infectious diseases were the main reasons for referrals and hospitaliziations of asylum seekers at the University Hospital Basel in both study periods with an increase of tropical infectious diseases and diseases associated to the living conditions of these patients in the current refugee crisis depending on the country of origin. A high percentage of hospitalized patients required isolation precautions.

The massive arrival of asylum seekers to Europe and Switzerland and their reception and procedure centres in the current refugee crisis has caused a considerable increase in the absolute number of asylum seeker referrals to the hospital. In 2014/15 a major shift to more refugees coming from the African (mainly from Eritrea) and Eastern Mediterranean Region (mainly from Syria) was observed due to formation of new crisis zones [3] [4] compared to mainly European refugees in 2004/05. The dominance of males and the young average age reflects the demographic pattern of asylum seekers in Switzerland [8,11] [13] [14].

The percentage of referred asylum seekers from the local reception and procedure centre to the hospital remained high and stable over the 2 study periods (in overall 711 of 8’697 asylum seekers; 8,2%). Considering the young age of the asylum seekers, the percentage of refugees seeking medical care is rather high indicating an increased demand for medical care in this population. Other explanations could be reduced access barriers or higher medical awareness of the asylum seekers.

Studies on health problems of asylum seekers in Switzerland in the years 2009 until 2013 report consistently rather low prevalences of infectious diseases in patients seeking medical advice [8] [10,11], ranging from 4.7 to 10%. Other studies indicate that chronic diseases and psychiatric conditions are the most important health problems [6,7]. This is in contrast to our findings. In our study infections were reported in around 20–30% of the referrals and were the most important reasons for hospitalization in both time periods which reaffirms recent reports of the increasing burden of communicable diseases in asylum seekers in the current refugee crisis [9] [15,16]. Interestingly, tropical and re-emerging infections diseases like malaria, louseborne relapsing fever or cutaneous diphtheria were rather frequent encountered in the current study period mainly due to ayslum seekers from endemic African countries.

Following reasons might explain the high rate of infectious diseases in our study population: First, the selection of patients referred from the reception and procedure centre just after arrival to Switzerland has enabled the detection of infectious diseases during the incubation period. Precarious hygienic conditions during the travel may facilitate the spread of infectious diseases [15] [17] being clinically still present upon arrival. Second, a referral bias is likely as minor health problems are managed by a primary care physician. Third, the suspicion of certain communicable diseases like e.g. tuberculosis with consecutive referral to the hospital may have led to an overrepresentation of infectious diseases. Fourth, improved diagnostic facilities (e.g. GenXpert for tuberculosis) enable the diagnosis of various infectious diseases more easily. Fifth, the massive arrival from mainly African refugees during spring and summer 2015 [18] [19] might have led to more dense and difficult living conditions of asylum seekers during travel and in the local reception and procedure centre with increased risk of transmission of highly contagious diseases such as varicella and influenza virus infection, meningococcal meningitis, scabies and skin infections with *Corynebacterium diphtheriae* or Methicillin-resistant *Staphylococcus aureus* (MRSA).

During both study periods almost 30% of admitted patients required isolation precautions. Suspected or confirmed active tuberculosis was responsible for >90% of all isolation precautions in the first, but only for one third in the second study period. Instead, other contagious diseases associated with overcrowding and lack of hygiene during travel and accommodation after arrival were diagnosed, like e.g. influenza, scabies, chickenpox, or cutaneous diphtheria [20]. Notably, cutaneous diphtheria cases have been reported only recently in Switzerland in asylum seekers from tropical countries. A diphtheria epidemic in Switzerland and Germany was described with 20 cases from Northeast Africa and Syria including these 3 patients in 2015. Whole genome sequencing suggests that transmission occurred most likely outside Europe [21].

Our study has limitations: We examined referrals of asylum seekers from one of the 5 reception and procedure centres in Switzerland. Our findings might differ from data of other regions of Switzerland or other Western countries. The migration routes as well as the origin and the background of the refugees arriving to European countries are changing continuously, making wide generalization difficult. Furthermore, asylum seekers usually spend only the first weeks of their stay at the reception and procedure centre which may lead to an underestimation of chronic health problems. Patients with mainly psychiatric problems are frequently referred directly to a psychiatric health institution (not located at the University Hospital of Basel), thus this important patient group is likely to be underrepresented in our study. Finally, our study design was retrospective with potentially incomplete data mainly from the first study period.

Our study has also strengths. Switzerland’s admission system for refugees is well organized and provides access to medical care for all asylum seekers at the day of entry. A primary care physician in the reception and procedure centre acts as gate keeper, constantly screens all newly arrived asylum seekers for health problems supported by an electronic decision making instrument in 28 languages and refers sick patients to the University Hospital Basel if necessary. Although only one reception and procedure centre was investigated the total number of refugees and patients analysed was large.

In conclusion, the changing demography of asylum seekers arriving in Switzerland in the current refugee crisis has led to a shift in disease patterns with an increase of infectious and tropical diseases, and the re-emergence of neglected infections associated with living conditions and problems of overcrowding during travel. Physicians taking care of refugees—in particular in Emergency Departments—should be aware of these new challenges. Our study has implications for medical education and health policy makers to ensure a timely diagnosis and therapy as well as implementation of hygiene precautions and vaccinations to decrease the risk of transmission of infections such as tuberculosis, varicella, diphtheria or scabies.

## Supporting information

S1 Table Entries into the local reception and procedure centre of Basel. In this table, the number of asylum seekers and their countries of origin arriving to the local reception and procedure centre of Basel during the two study periods of 01. September 2004 until 31. August 2005 and 01. September 2014 until 31. August 2015 are specified. Additionally, the average stay of asylum seekers in the local reception and procedure centre is displayed for both study periods.
(DOCX)

S2 Table Countries of origin of the patients presenting at the University Hospital of Basel. This table summarises the origin of all patients (number [n] and percentage [%]) from the local reception and procedure centre of Basel presenting to the University Hospital of Basel and the origin of the subset of the hospitalized patients for both time periods (2004/05 and 2014/15) and for each time period 2004/05 and 2014/15 separately. 2004/05 includes the time period from 01. September 2004 until 31. August 2005 2014/15 includes the time period from 01. September 2014 until 31. August 2015.
(DOCX)

S3 Table Hospitalized asylum seekers with co-infections in 2004/05 (n = 1) and 2014/15 (n = 12). AFR: African Region; WPR: Western Pacific Region; MRSA: Methicillin-resistant *Staphylococcus aureus*.
(DOCX)
