# Supplementary material for: Increasing prevalence of infectious diseases in asylum seekers at a tertiary care hospital in Switzerland
Source: PLoS One. 2017 Jun 15;12(6):e0179537. doi: 10.1371/journal.pone.0179537 (PMC5472310; doi:10.1371/journal.pone.0179537)
Supplement: S1 Table — In this table, the number of asylum seekers and their countries of origin arriving to the local reception and procedure centre of Basel during the two study periods of 01. September 2004 until 31. August 2005 and 01. September 2014 until 31. August 2015 are specified. Additionally, the average stay of asylum seekers in the local reception and procedure centre is displayed for both study periods. (DOCX) [file pone.0179537.s001.docx]

**Supporting information**

**S1 Table: Entries into the local reception and procedure centre of Basel**

| **Total (n, %)**  **01.09.2004 until 31.08.2005** | **2454** | **100%** |  | **Total (n, %)**  **01.09.2014 until 31.08.2015** | **6243** | **100%** |
| --- | --- | --- | --- | --- | --- | --- |
| Turkey | 416 | 17.0% |  | Eritrea | 2426 | 38.9% |
| Serbia | 310 | 12.6% |  | Syria | 801 | 12.8% |
| Bulgaria | 275 | 11.2% |  | Sri Lanka | 572 | 9.2% |
| Russia | 152 | 6.2% |  | Afghanistan | 298 | 4.8% |
| Iraq | 127 | 5.2% |  | Albania | 217 | 3.5% |
| Georgia | 114 | 4.6% |  | Iraq | 182 | 2.9% |
| Sri Lanka | 72 | 2.9% |  | Kosovo | 138 | 2.2% |
| unknown origin | 67 | 2.7% |  | China | 126 | 2.0% |
| Iran | 61 | 2.5% |  | Somalia | 126 | 2.0% |
| Nigeria | 55 | 2.2% |  | Macedonia | 125 | 2.0% |
| Bangladesh | 51 | 2.1% |  | Turkey | 108 | 1.7% |
| Afghanistan | 48 | 2.0% |  | Ethiopia | 98 | 1.6% |
| Nepal | 48 | 2.0% |  | Serbia | 95 | 1.5% |
| Armenia | 45 | 1.8% |  | Bosnia-Herzegovina | 91 | 1.5% |
| Macedonia | 37 | 1.5% |  | no nationality | 58 | 0.9% |
| Algeria | 35 | 1.4% |  | unknown origin | 54 | 0.9% |
| Bosnia-Herzegovina | 35 | 1.4% |  | Georgia | 51 | 0.8% |
| no nationality | 29 | 1.2% |  | Nigeria | 49 | 0.8% |
| India | 28 | 1.1% |  | Algeria | 44 | 0.7% |
| Somalia | 27 | 1.1% |  | Sudan | 41 | 0.7% |
| Cameroon | 25 | 1.0% |  | Russia | 41 | 0.7% |
| Belarus | 22 | 0.9% |  | Hungary | 39 | 0.6% |
| China | 22 | 0.9% |  | Morocco | 38 | 0.6% |
| DR of the Congo | 22 | 0.9% |  | Iran | 37 | 0.6% |
| Albania | 21 | 0.9% |  | Gambia | 36 | 0.6% |
| Ukraine | 21 | 0.9% |  | Ukraine | 33 | 0.5% |
| Pakistan | 20 | 0.8% |  | Tunisia | 32 | 0.5% |
| Tunisia | 19 | 0.8% |  | Pakistan | 27 | 0.4% |
| Romania | 18 | 0.7% |  | Senegal | 20 | 0.3% |
| Syria | 17 | 0.7% |  | Libya | 20 | 0.3% |
| Azerbaijan | 15 | 0.6% |  | Guinea | 18 | 0.3% |
| Moldovia | 15 | 0.6% |  | Azerbaijan | 12 | 0.2% |
| Angola | 13 | 0.5% |  | Cameroon | 10 | 0.2% |
| Togo | 13 | 0.5% |  | Togo | 10 | 0.2% |
| Ivory Coast | 11 | 0.4% |  | Egypt | 10 | 0.2% |
| Liberia | 10 | 0.4% |  | Montenegro | 9 | 0.1% |
| Mongolia | 10 | 0.4% |  | Mali | 9 | 0.1% |
| Libya | 8 | 0.3% |  | USA | 8 | 0.1% |
| Sierra Leone | 8 | 0.3% |  | Bangladesh | 8 | 0.1% |
| Sudan | 8 | 0.3% |  | DR of the Congo | 8 | 0.1% |
| Eritrea | 7 | 0.3% |  | Mongolia | 8 | 0.1% |
| Latvia | 7 | 0.3% |  | Armenia | 8 | 0.1% |
| Libanon | 7 | 0.3% |  | Moldovia | 6 | 0.1% |
| Ethiopia | 6 | 0.2% |  | Ghana | 6 | 0.1% |
| Kroatien | 6 | 0.2% |  | Ivory coast | 5 | 0.1% |
| Burundi | 5 | 0.2% |  | Benin | 5 | 0.1% |
| Kazakhstan | 5 | 0.2% |  | Guinea-Bissau | 4 | 0.1% |
| Litvania | 5 | 0.2% |  | Germany | 4 | 0.1% |
| Guinea | 4 | 0.2% |  | South Korea | 4 | 0.1% |
| Mali | 4 | 0.2% |  | Mauretania | 4 | 0.1% |
| Morocco | 4 | 0.2% |  | Tschad | 3 | 0.0% |
| Myanmar | 4 | 0.2% |  | Belgium | 3 | 0.0% |
| Uganda | 3 | 0.1% |  | Uganda | 3 | 0.0% |
| Zimbabwe | 3 | 0.1% |  | Nepal | 3 | 0.0% |
| Germany | 2 | 0.1% |  | Latvia | 3 | 0.0% |
| Ghana | 2 | 0.1% |  | Croatia | 2 | 0.0% |
| Jordan | 2 | 0.1% |  | Italia | 2 | 0.0% |
| Kenia | 2 | 0.1% |  | Angola | 2 | 0.0% |
| Colombia | 2 | 0.1% |  | Niger | 2 | 0.0% |
| Congo | 2 | 0.1% |  | Uzbekistan | 2 | 0.0% |
| Cuba | 2 | 0.1% |  | Kazakhstan | 2 | 0.0% |
| Mauritania | 2 | 0.1% |  | Liberia | 2 | 0.0% |
| Niger | 2 | 0.1% |  | Poland | 2 | 0.0% |
| South Africa | 2 | 0.1% |  | Belarus | 2 | 0.0% |
| Uzbekistan | 2 | 0.1% |  | Romania | 2 | 0.0% |
| Equatorial Guinea | 1 | 0.0% |  | Jemen | 2 | 0.0% |
| Benin | 1 | 0.0% |  | Kongo | 2 | 0.0% |
| Burkina Faso | 1 | 0.0% |  | Slovakia | 2 | 0.0% |
| Canada | 1 | 0.0% |  | Jordan | 2 | 0.0% |
| No information | 1 | 0.0% |  | Mexico | 1 | 0.0% |
| Rwanda | 1 | 0.0% |  | Bhutan | 1 | 0.0% |
| Senegal | 1 | 0.0% |  | Sierra Leone | 1 | 0.0% |
| Slovakia | 1 | 0.0% |  | Kyrgyzstan | 1 | 0.0% |
| Thailand | 1 | 0.0% |  | Myanmar | 1 | 0.0% |
| Chad | 1 | 0.0% |  | Venezuela | 1 | 0.0% |
| Hungary | 1 | 0.0% |  | Burkina Faso | 1 | 0.0% |
| USA | 1 | 0.0% |  | Great Britain | 1 | 0.0% |
|  |  |  |  | Lithuania | 1 | 0.0% |
|  |  |  |  | Colombia | 1 | 0.0% |
|  |  |  |  | India | 1 | 0.0% |
|  |  |  |  | Vietnam | 1 | 0.0% |
|  |  |  |  | France | 1 | 0.0% |
|  |  |  |  | Guatemala | 1 | 0.0% |
|  |  |  |  | Bulgaria | 1 | 0.0% |
|  |  |  |  | Netherlands | 1 | 0.0% |
|  |  |  |  | Bahrain | 1 | 0.0% |
|  |  |  |  | Rwanda | 1 | 0.0% |
|  |  |  |  | Tansania | 1 | 0.0% |
|  |  |  |  | Slovenia | 1 | 0.0% |
|  |  |  |  | Libanon | 1 | 0.0% |
| **Average stay in days** | **20.1** |  |  | **Average stay in days** | **28.4** |  |
