# Supplementary material for: Increasing prevalence of infectious diseases in asylum seekers at a tertiary care hospital in Switzerland
Source: PLoS One. 2017 Jun 15;12(6):e0179537. doi: 10.1371/journal.pone.0179537 (PMC5472310; doi:10.1371/journal.pone.0179537)
Supplement: S2 Table — This table summarises the origin of all patients (number [n] and percentage [%]) from the local reception and procedure centre of Basel presenting to the University Hospital of Basel and the origin of the subset of the hospitalized patients for both time periods (2004/05 and 2014/15) and for each time period 2004/05 and 2014/15 separately. 2004/05 includes the time period from 01. September 2004 until 31. August 2005 2014/15 includes the time period from 01. September 2014 until 31. August 2015. (DOCX) [file pone.0179537.s002.docx]

**Supporting information**

**S2 Table: Countries of origin of the patients presenting at the University Hospital of Basel**

|  | **All patients** | | | | | | **Hospitalized patients** | | | | | |
| --- | --- | --- | --- | --- | --- | --- | --- | --- | --- | --- | --- | --- |
|  | **both periods** | | **2004/05** | | **2014/15** | | **both periods** | | **2004/05** | | **2014/15** | |
|  | n | % | n | % | n | % | n | % | n | % | n | % |
| **Total** | **711** | **100** | **195** | **100** | **516** | **100** | **126** | **100** | **45** | **100** | **81** | **100.0** |
| **European Region** | 126 | 17.7 | 66 | 33.8 | 60 | 11.6 | 38 | 30.2 | 25 | 55.6 | 13 | 16.0 |
| **Turkey** | 27 | 3.8 | 27 | 13.8 | 0 | 0.0 | 6 | 4.8 | 6 | 13.3 | 0 | 0.0 |
| **Bulgaria** | 15 | 2.1 | 15 | 7.7 | 0 | 0.0 | 7 | 5.6 | 7 | 15.6 | 0 | 0.0 |
| **Kosovo** | 15 | 2.1 | 0 | 0.0 | 15 | 2.9 | 2 | 1.6 | 0 | 0.0 | 2 | 2.5 |
| **Georgia** | 12 | 1.7 | 6 | 3.1 | 6 | 1.2 | 2 | 1.6 | 1 | 2.2 | 1 | 1.2 |
| **Serbia** | 12 | 1.7 | 4 | 2.1 | 8 | 1.6 | 4 | 3.2 | 3 | 6.7 | 1 | 1.2 |
| **Albania** | 10 | 1.4 | 4 | 2.1 | 6 | 1.2 | 3 | 2.4 | 2 | 4.4 | 1 | 1.2 |
| **Russia** | 7 | 1.0 | 0 | 0.0 | 7 | 1.4 | 4 | 3.2 | 0 | 0.0 | 4 | 4.9 |
| **Macedonia** | 6 | 0.8 | 1 | 0.5 | 5 | 1.0 | 3 | 2.4 | 1 | 2.2 | 2 | 2.5 |
| **Belarus** | 4 | 0.6 | 4 | 2.1 | 0 | 0.0 | 3 | 2.4 | 2 | 4.4 | 1 | 1.2 |
| **Bosnia-Herzegovina** | 4 | 0.6 | 0 | 0.0 | 4 | 0.8 | 0 | 0.0 | 0 | 0.0 | 0 | 0.0 |
| **Armenia** | 3 | 0.4 | 1 | 0.5 | 2 | 0.4 | 0 | 0.0 | 0 | 0.0 | 0 | 0.0 |
| **Hungary** | 2 | 0.3 | 0 | 0.0 | 2 | 0.4 | 0 | 0.0 | 0 | 0.0 | 0 | 0.0 |
| **Lithuania** | 2 | 0.3 | 0 | 0.0 | 2 | 0.4 | 0 | 0.0 | 0 | 0.0 | 0 | 0.0 |
| **Azerbaijan** | 1 | 0.1 | 1 | 0.5 | 0 | 0.0 | 0 | 0.0 | 0 | 0.0 | 0 | 0.0 |
| **Chechenia** | 1 | 0.1 | 1 | 0.5 | 0 | 0.0 | 1 | 0.8 | 1 | 2.2 | 0 | 0.0 |
| **Croatia** | 1 | 0.1 | 1 | 0.5 | 0 | 0.0 | 1 | 0.8 | 1 | 2.2 | 0 | 0.0 |
| **Moldavia** | 1 | 0.1 | 1 | 0.5 | 0 | 0.0 | 1 | 0.8 | 1 | 2.2 | 0 | 0.0 |
| **Romania** | 1 | 0.1 | 0 | 0.0 | 1 | 0.2 | 1 | 0.8 | 0 | 0.0 | 1 | 1.2 |
| **Slovakia** | 1 | 0.1 | 0 | 0.0 | 1 | 0.2 | 0 | 0.0 | 0 | 0.0 | 0 | 0.0 |
| **Ukraine** | 1 | 0.1 | 0 | 0.0 | 1 | 0.2 | 0 | 0.0 | 0 | 0.0 | 0 | 0.0 |
| **Eastern Mediterranean Region** | 103 | 14.5 | 25 | 12.8 | 78 | 15.1 | 19 | 15.1 | 7 | 15.6 | 12 | 14.8 |
| **Syria** | 22 | 3.1 | 0 | 0.0 | 22 | 4.3 | 8 | 6.3 | 0 | 0.0 | 8 | 9.9 |
| **Iraq** | 19 | 2.7 | 12 | 6.2 | 7 | 1.4 | 2 | 1.6 | 2 | 4.4 | 0 | 0.0 |
| **Somalia** | 15 | 2.1 | 0 | 0.0 | 15 | 2.9 | 1 | 0.8 | 0 | 0.0 | 1 | 1.2 |
| **Afghanistan** | 14 | 2.0 | 2 | 1.0 | 12 | 2.3 | 0 | 0.0 | 0 | 0.0 | 0 | 0.0 |
| **Tunisia** | 10 | 1.4 | 0 | 0.0 | 10 | 1.9 | 0 | 0.0 | 0 | 0.0 | 0 | 0.0 |
| **Morocco** | 8 | 1.1 | 2 | 1.0 | 6 | 1.2 | 2 | 1.6 | 1 | 2.2 | 1 | 1.2 |
| **Libya** | 7 | 1.0 | 4 | 2.1 | 3 | 0.6 | 4 | 3.2 | 2 | 4.4 | 2 | 2.5 |
| **Sudan** | 5 | 0.7 | 2 | 1.0 | 3 | 0.6 | 1 | 0.8 | 1 | 2.2 | 0 | 0.0 |
| **West Bank** | 2 | 0.3 | 2 | 1.0 | 0 | 0.0 | 1 | 0.8 | 1 | 2.2 | 0 | 0.0 |
| **Pakistan** | 1 | 0.1 | 1 | 0.5 | 0 | 0.0 | 0 | 0.0 | 0 | 0.0 | 0 | 0.0 |
| **South-East Asia Region** | 25 | 3.5 | 3 | 1.5 | 22 | 4.3 | 4 | 3.2 | 0 | 0.0 | 4 | 4.9 |
| **Sri Lanka** | 23 | 3.2 | 2 | 1.0 | 21 | 4.1 | 3 | 2.4 | 0 | 0.0 | 3 | 3.7 |
| **Bangladesh** | 1 | 0.1 | 0 | 0.0 | 1 | 0.2 | 1 | 0.8 | 0 | 0.0 | 1 | 1.2 |
| **India** | 1 | 0.1 | 1 | 0.5 | 0 | 0.0 | 0 | 0.0 | 0 | 0.0 | 0 | 0.0 |
| **Western Pacific Region** | 11 | 1.5 | 4 | 2.1 | 7 | 1.4 | 5 | 4.0 | 2 | 4.4 | 3 | 3.7 |
| **China (incl. Tibet)** | 10 | 1.4 | 3 | 1.5 | 7 | 1.4 | 4 | 3.2 | 1 | 2.2 | 3 | 3.7 |
| **Mongolia** | 1 | 0.1 | 1 | 0.5 | 0 | 0.0 | 1 | 0.8 | 1 | 2.2 | 0 | 0.0 |
| **African Region** | 177 | 24.9 | 8 | 4.1 | 169 | 32.8 | 46 | 36.5 | 2 | 4.4 | 44 | 54.3 |
| **Eritrea** | 154 | 21.7 | 0 | 0.0 | 154 | 29.8 | 43 | 34.1 | 0 | 0.0 | 43 | 53.1 |
| **Nigeria** | 7 | 1.0 | 5 | 2.6 | 2 | 0.4 | 0 | 0.0 | 0 | 0.0 | 0 | 0.0 |
| **Algeria** | 4 | 0.6 | 2 | 1.0 | 2 | 0.4 | 1 | 0.8 | 1 | 2.2 | 0 | 0.0 |
| **Guinea** | 3 | 0.4 | 0 | 0.0 | 3 | 0.6 | 0 | 0.0 | 0 | 0.0 | 0 | 0.0 |
| **Ghana** | 3 | 0.4 | 1 | 0.5 | 2 | 0.4 | 1 | 0.8 | 1 | 2.2 | 0 | 0.0 |
| **Sierra Leone** | 2 | 0.3 | 0 | 0.0 | 2 | 0.4 | 0 | 0.0 | 0 | 0.0 | 0 | 0.0 |
| **Ethiopia** | 2 | 0.3 | 0 | 0.0 | 2 | 0.4 | 0 | 0.0 | 0 | 0.0 | 0 | 0.0 |
| **Gambia** | 1 | 0.1 | 0 | 0.0 | 1 | 0.2 | 1 | 0.8 | 0 | 0.0 | 1 | 1.2 |
| **Uganda** | 1 | 0.1 | 0 | 0.0 | 1 | 0.2 | 0 | 0.0 | 0 | 0.0 | 0 | 0.0 |
| **Region of the Americas** | 8 | 1.1 | 0 | 0.0 | 8 | 1.6 | 3 | 2.4 | 0 | 0.0 | 3 | 3.7 |
| **United States of America** | 5 | 0.7 | 0 | 0.0 | 5 | 1.0 | 3 | 2.4 | 0 | 0.0 | 3 | 3.7 |
| **French Guyana** | 3 | 0.4 | 0 | 0.0 | 3 | 0.6 | 0 | 0.0 | 0 | 0.0 | 0 | 0.0 |
| **unknown** | 261 | 36.7 | 89 | 45.6 | 172 | 33.3 | 11 | 8.7 | 9 | 20.0 | 2 | 2.5 |
