# Supplementary material for: Increasing prevalence of infectious diseases in asylum seekers at a tertiary care hospital in Switzerland
Source: PLoS One. 2017 Jun 15;12(6):e0179537. doi: 10.1371/journal.pone.0179537 (PMC5472310; doi:10.1371/journal.pone.0179537)
Supplement: S3 Table — AFR: African Region; WPR: Western Pacific Region; MRSA: Methicillin-resistant Staphylococcus aureus. (DOCX) [file pone.0179537.s003.docx]

**Supporting information**

**S3 Table**: Hospitalized asylum seekers with co-infections in 2004/05 (n=1) and 2014/15 (n=12)

| **Main infectious diagnosis** | **Co-infections** | **Origin** |
| --- | --- | --- |
| **2004/05** |  |  |
| Suspected tuberculosis (not confirmed) | Erythema chronicum migrans | WPR |
| **2014/15** |  |  |
| Cutaneous bacterial abscess | Scabies | AFR |
| Cutaneous bacterial abscess | Scabies | AFR |
| Active pulmonary tuberculosis | Viral respiratory infection | WPR |
| Influenza B with bacterial superinfection | HIV CDC B3 (initial diagnosis) | AFR |
| Malaria | Scabies | AFR |
| Varicella virus infection | Colonisation with MRSA | AFR |
| Fungal pulmonary infection | Scabies | AFR |
| Cutaneous diphtheria | Colonisation with MRSA, scabies | AFR |
| Varicella virus infection | Colonisation with MRSA | AFR |
| Viral respiratory infection | Scabies, cutaneous diphtheria | AFR |
| Malaria | Scabies | AFR |
| Viral respiratory infection | Scabies, cutaneous diphtheria | AFR |
